# Probiotics: Reiterating What They Are and What They Are Not

**DOI:** 10.3389/fmicb.2019.00424

**Published:** 2019-03-12

**Authors:** Gregor Reid, Azza A. Gadir, Raja Dhir

**Affiliations:** ^1^Canadian R&D Centre for Human Microbiome and Probiotics, Lawson Health Research Institute, London, ON, Canada; ^2^Departments of Microbiology and Immunology and Surgery, Western University, London, ON, Canada; ^3^Division of Immunology, Boston Children’s Hospital, Boston, MA, United States; ^4^Department of Pediatrics, Harvard Medical School, Boston, MA, United States; ^5^Seed, Los Angeles, CA, United States

**Keywords:** human microbiome, probiotics, scientific definition, stewardship, requirements, human health, microbial therapies, translational microbiology

## Abstract

It has been over seventeen years since the scientific definition of probiotics was crafted, along with guidelines ensuring the appropriate use of the term. This definition is now used globally, yet on a consistent basis scientists, media and industry misrepresent probiotics or make generalized statements that illustrate a misunderstanding of their utility and limitations. The rate of discovery of novel organisms with potentially therapeutic benefit for both human and environmental health is progressing at an unprecedented rate. However, the term “probiotic” is often misapplied to describe any microbe with plausible therapeutic utility in the human host. It is argued that strict compliance to the scientific definition of the term “probiotic” and avoidance of generalizations to the whole field of probiotics based upon studies of one product, will help advance the development and validation of microbial therapies, and applications to improve human health.

## Introduction

In 2001, an Expert Panel was convened at the request of the Food and Agriculture Organization of the United Nations, and supported by the World Health Organization, to clarify the definition of probiotics. The resultant publication defined probiotics as “Live microorganisms which when administered in adequate amounts confer a health benefit on the host” (FAO/WHO, 2002). In 2014, a consensus panel reiterated the definition, with one small change replacing “which” with “that” (Hill et al., 2014).

Since 2001, scientific investigation of probiotics has grown substantially. As of February 11, 2019, there were 20,315 papers indexed to the term “probiotic” compared to 760 papers prior to 2001. Commercially, the sales of probiotics are over $40 billion and are projected to reach over $64 billion by 2023 (Global Market Insights, 2016).

The probiotic field represents the translational potential of microbiology to humans and animals. Despite well characterized mechanisms for microbial therapies to affect a number of organ systems in the host, confusion persists around the precise conditions necessary for a single microbe or microbial consortia to claim probiotic effect (verb) or qualify as a probiotic (noun). Specifically, a set of guidelines stipulates the need for both microbial strain designation and for at least one human study to be performed on the target host to be considered probiotic (FAO/WHO, 2002). Liberal and loose usage of the term has resulted in confusion and as a result, probiotics have collectively garnered a degree of global skepticism. The aim of this article is to clarify the term probiotics and urge researchers to employ precision and consistency in both their investigations and communication of data pertaining to microbes for human health.

## Illustrating the Problems

A physician wanting to provide a patient with advice on preventing traveler’s diarrhea (TD) may have referenced a recent meta-analysis by Bae (2018) regarding the use of probiotics as a treatment. This publication used a seemingly appropriate inclusion criteria of double-blind, placebo-controlled, randomized human trials with TD as an outcome, and various statistical methods to conclude that probiotics were efficacious in preventing TD. However, a closer look at the listed interventions shows one study that utilized dead lactobacilli and two other studies that included the administration of prebiotics. Caution must be exercised when interpreting such data, given that probiotics must be live (FAO/WHO, 2002; Sanders et al., 2007), and the definition of a prebiotic is “a substrate that is selectively utilized by host microorganisms conferring a health benefit” (Gibson et al., 2017).

Confusion within the field is not restricted to meta-analyses. Conclusions are often advanced based on improperly designed intervention studies that extrapolate broad generalizations from a limited data set. Several examples are worth citing. One study concentrating on the use of probiotics in acute pancreatitis suggested that they contributed to bacterial translocation and enterocyte damage in organ failure (Besselink et al., 2004, 2009). Yet, there is no direct evidence that the administered strains were the cause (Reid et al., 2008). Aside from the issue of whether or not this is an appropriate clinical setting to administer extrinsic living bacteria, the study’s conclusion has been enlarged to imply that any or all probiotics might adversely impact all patients with pancreatitis. Nine years later, that initial study has been fully reassessed concluding that probiotics are not contraindicated in pancreatitis. The prior negative outcomes were related to unexpected lactic acidosis from the fermentation of carbohydrates (Bongaerts and Severijnen, 2016). Indeed, this more recent study recommended starting probiotics immediately upon diagnosis, massively increasing the probiotic dose, and limiting fermentable carbohydrates.

To avoid unwarranted damage to the reputation of probiotics, stringent testing and careful clinical guidelines are imperative in the treatment and management of high risk adults and children (Sanders et al., 2016). Not all centers have avoided use of probiotics in seriously ill patients as a result of the pancreatitis paper, and indeed one site has reported substantial benefits in treating seriously ill trauma patients with probiotics or synbiotics (Giamarellos-Bourboulis et al., 2009). Furthermore, there is clear evidence of probiotics as useful in preventing necrotizing enterocolitis in premature, low-birth weight babies which is certainly a critically ill patient sub-set (Patel and Underwood, 2018).

Another pertinent example of the over interpretation of limited data is a recent study conducted on 34 patients suffering from “brain fogginess,” which is a set of presentations including impaired concentration, confusion, and poor short-term memory (Rao et al., 2018). Based on the observation that consumption of fermented food or commercially available microbial products was a shared trait amongst the patients reporting brain fog, the study implied that all probiotics could be causative agents of brain fogginess. While the definition of probiotics accommodates strains of any taxa, it is unlikely that these included the *Streptococcus*, *Staphylococcus*, *Neisseria*, or *Hemophilus*, which were the primary organisms detected by culture in the duodenal aspirates. Furthermore, 68% of the cohort presented with small intestinal bacterial overgrowth (SIBO) and there were no data to verify patients were acidotic; a crucial linkage considering many lactobacilli and all bifidobacteria only produce L-lactate and not D-lactate (Vitetta et al., 2017; Petrova et al., 2018; Quigley et al., 2018). In the future, appropriate trial design can strengthen the field by moving away from observational and correlative data sets. In this case, the study concluded that probiotics may be causative of brain fog on the observation that antibiotic therapy alleviated symptoms of brain fog, whereas a more appropriately designed study would have split the patients after antibiotic therapy into two groups, and upon reintroduction of probiotics in a defined cohort, compared the emergence of symptoms against the antibiotic-treated control.

In a third example, a recent study suggesting probiotics impair naïve microbiota recovery after antibiotic therapy concluded that probiotics may be harmful to the gut microbiota when administered after antibiotic use (Suez et al., 2018). In this instance, Suez et al. (2018) performed an intervention study on healthy subjects given high doses of metronidazole and ciprofloxacin. The subjects received evacuation therapy twice in 28 days so that intestinal samples could be acquired. Based on the observation that 10–15 species had a longer recovery time compared to spontaneous recovery and autologous fecal microbiota transplantation (aFMT), the study put forward the thesis that probiotic consumption may present latent danger to the gut microbiome and, by extension, the host. There are serious limitations to asserting broad generalizations from one narrow data set to the field as a whole. In this case, two subsequent human studies published within a month of the Suez et al. (2018) study and a second report from that group (Zmora et al., 2018) presented alternative data (De Wolfe et al., 2018; Korpela et al., 2018). The first demonstrated that probiotic supplementation restored normal microbiota composition and function in antibiotic-treated and cesarean-born infants. A second study examined a different microbial preparation and its effect on microbiome recovery after antibiotic therapy (De Wolfe et al., 2018). Notably, the microbial intervention contained a 700% higher dosage and was administered for twice the intervention period as the Suez et al. (2018) intervention with opposite data showing improved microbiome alpha-diversity in the “probiotics” group as measured by the Shannon diversity index, a heterogeneity measure that combines richness and evenness components of microbial diversity (De Wolfe et al., 2018). It is worth noting that all three publications were released in the same month, yet the results of one were extrapolated to the field as a whole, hindering an open scientific dialog on the advantages or limitations of probiotics or their reproducible effects based on strain specificity.

Relevantly, it is worth nothing that one should not rely on metagenomic and 16s rRNA data as an endpoint alone, but rather monitor clinical and functional outcomes, for example an increase in short-chain fatty acid production and their direct immunological effect on colonic Treg cells (Smith et al., 2013) and other pathological processes (Cox et al., 2009; Ho et al., 2018; Nagpal et al., 2018). Another effect of well characterized probiotic strains is to confirm protection of the intestinal epithelium, in some cases reversing damage caused by antibiotics (Resta-Lenert and Barrett, 2006).

## The Minimal Requirements for the Evaluation of an Effective Probiotic

As indicated in the original FAO/WHO (2002) report, there are certain expectations required to call an organism “probiotic.” These have been further clarified in 2014 (Hill et al., 2014), and clearly must include:

The definition of probiotics as “Live microorganisms, that when administered in adequate amounts, confer a health benefit on the host.”

1. That microbes must be alive in an adequate number when administered.
2. Strains must be identified genetically, classified using the latest terminology, and designated by numbers, letters, or names.
3. Appropriately sized and designed studies must be performed to designate a strain as probiotic and using the strain(s) on the host to which the probiotics are intended (human, livestock, companion animal, etc).
4. Strains shown to confer a benefit for one condition may not be probiotic for another application.
5. Strains that are probiotic for humans but are being used in animal studies should be clearly designated as human probiotics under experimental testing.

Fermented foods, prebiotics, fecal microbiota transplant, and microbial strains of the same genus or species as documented probiotic strains but have not undergone appropriate testing on the target host should not be considered as probiotics based on adherence to the scientific definition.

## Guidance for Physicians

Within the field of microbiome science, the rate of discovery of novel organisms with potentially therapeutic benefit for the human host is progressing rapidly. Emerging research on microbial strains includes some with systemic immunomodulatory activity (Geva-Zatorsky et al., 2017), the prevention and treatment of food allergies (Kim et al., 2017; Feehley et al., 2019), modulation of the gut-liver axis (Bajaj, 2019), production of neuroactive metabolites (Valles-Colomer et al., 2019), and inhibitory activity against infectious microbes in the gut (Kau et al., 2011), skin (Kober and Bowe, 2015), and urogenital tract (Gottschick et al., 2017). Furthermore, microbes are now understood as integral to an extensive number of essential metabolic processes (Table 1) (Hentzer and Givskov, 2003; Ley et al., 2006; Abubucker et al., 2012; Karlsson et al., 2012; Morgan et al., 2012; den Besten et al., 2013; Tang et al., 2013; Tang and Hazen, 2014; Fiorucci and Distrutti, 2015; Hartstra et al., 2015; Magnúsdóttir et al., 2015; McCabe et al., 2015; Neuman et al., 2015; O’Mahony et al., 2015; Yano et al., 2015; Savidge, 2016; Sonnenburg and Bäckhed, 2016; Thaiss et al., 2016; Yan et al., 2016; Zeng et al., 2016; Blander et al., 2017; Koppel et al., 2017; Weiss and Hennet, 2017; Desselberger, 2018; Fukui et al., 2018; Schirmer et al., 2018; Zhang et al., 2018). From these discoveries will emerge new microbial-based interventions.

**Table 1:** Physiological and metabolic processes influenced by the human microbiome.

Processes	Reference
Enzymatic pathways, glycosaminoglycan degradation	Abubucker et al., 2012; Koppel et al., 2017
Energy metabolism (short chain fatty acids, glucose)	den Besten et al., 2013; Hartstra et al., 2015; Sonnenburg and Bäckhed, 2016
Neurotransmitter production	O’Mahony et al., 2015; Yano et al., 2015; Savidge, 2016
Vitamin absorption	Magnúsdóttir et al., 2015
Regulation of bile acid metabolism, (deoxycholic and lithocholic acids, bile salts)	Fiorucci and Distrutti, 2015; Weiss and Hennet, 2017
Endocrine and gut hormone regulation	Neuman et al., 2015; Fukui et al., 2018
Adaptive immunity, mucosal and systemic immunity	Thaiss et al., 2016; Desselberger, 2018
Cell proliferation, mucosal barrier protection, inflammation	Morgan et al., 2012; Blander et al., 2017; Schirmer et al., 2018
Protection against pathogens	Zeng et al., 2016; Zhang et al., 2018
Vascularization, tri-ethylamine associated atherosclerosis, tri-ethylamine N-oxide (TMAO) production	Karlsson et al., 2012; Tang et al., 2013; Tang and Hazen, 2014
Bone mass	McCabe et al., 2015; Yan et al., 2016
Appetite signaling, obesity	Hentzer and Givskov, 2003; Ley et al., 2006
Metabolic transformation of xenobiotics (small molecules foreign to the body)	Koppel et al., 2017


Therefore, it is expected that the misuse of the term “probiotic” to describe any microbe with plausible therapeutic utility in the human host may persist unless there is strict adherence to the requirements of an exacting definition. To the extent that physicians may require guidance for evaluating the merits of current and future microbial preparations, three key elements in determining the validity of a probiotic are proposed:

1. Evidence that the strain(s) has been tested in a randomized, controlled or equivalent human trial, in either a heterogeneous population or stratified based on defined characteristics of host or microbial genomics.
2. The dose and viability in the product are equal to that of the human trial(s).
3. Whole genome strain characterization and transparently declared strain designation are provided.

If a strain is selected for a specific mechanism, such as being able to upregulate gut barrier proteins, and is then tested in humans and shown to improve gut barrier function leading to a health benefit (Iemoli et al., 2012), then inclusion of that strain at the tested dose would be expected to convey the same outcome even in the presence of other strains. An example is *B. breve* BR03 being selected for immunomodulatory and gut barrier function activities and consequently being found in a 300-person study to increase Treg cells, or in 49 children with Celiac disease to decrease TNFα production (Del Piano et al., 2010; Klemenak et al., 2015).

Studies of the human microbiome have established that despite significant interpersonal variation at the species level, many core metabolic functions are maintained amongst individuals in a population (Bogiatzi et al., 2018; Kleerebezem et al., 2018; Wandro et al., 2018). Therefore, as the field of personalized medicine gains traction, it is important that proponents of individualized therapies clearly define the basis for population stratification, and demonstrate efficacy for the proposed personalized therapy in the specific subpopulation being targeted. A more universal approach would entail the discovery and validation of microbes with significant and reproducible effects across a heterogeneous population. Therapies under both a stratified and heterogeneous population would meet the guidelines of probiotic, provided the impact on human health is established in a controlled human trial.

## Future Directions

Research investigating microbial administration to, or modulation of, the human microbiome to improve health has been progressing at a rapid rate since the term probiotics was defined in 2001. It has been amplified by the Human Microbiome Project and sustained by the interdisciplinary efforts beyond microbiology that have contributed to the velocity of microbiome research worldwide. It comes at a time where there has never been a greater appreciation for the microbes that inhabit life-forms and the planet. The potential to manipulate these microbial ecosystems offers great hope for new preventive and treatment options for many diseases. The idea that ingesting or administering live microbes can serve therapeutic functions is gaining prominence, but just as too many microbiome studies in rodents and humans “oversell” their limited findings (Hooks et al., 2018), too much misinformation has stemmed from misuse of the probiotic term. It is hoped that the fundamental principles outlined herein, that encourage adherence to a strict scientific definition of “probiotics” and rigorous assessment of their actual clinical outcomes will assert greater clarity and foster a deeper understanding within this dynamic field.

## Author Contributions

GR and RD conceived and wrote the original version of the manuscript. GR, RD, and AG revised the manuscript to meet the reviewers’ comments.

## Conflict of Interest Statement

RD is a founder in Seed, a biotechnology company developing microbial therapies not discussed in this paper. GR is a scientific advisor to Seed on probiotics. AG is involved in research and development at Seed and developing intellectual property related to microbial regulation of immune mechanisms underlying food allergies, which is not discussed in this paper.
